# An Assessment of Surface Treatments for Adhesion of Polyimide Thin Films

**DOI:** 10.3390/polym13121955

**Published:** 2021-06-12

**Authors:** Marco Cen-Puc, Andreas Schander, Minerva G. Vargas Gleason, Walter Lang

**Affiliations:** Institute for Microsensors, Actuators and Systems (IMSAS), University of Bremen, 28359 Bremen, Germany; mvargas@imsas.uni-bremen.de (M.G.V.G.); wlang@imsas.uni-bremen.de (W.L.)

**Keywords:** polyimide films, plasma treatment, surface modification, surface wettability, adhesion, peel strength

## Abstract

Polyimide films are currently of great interest for the development of flexible electronics and sensors. In order to ensure a proper integration with other materials and PI itself, some sort of surface modification is required. In this work, microwave oxygen plasma, reactive ion etching oxygen plasma, combination of KOH and HCl solutions, and polyethylenimine solution were used as surface treatments of PI films. Treatments were compared to find the best method to promote the adhesion between two polyimide films. The first selection of the treatment conditions for each method was based on changes in the contact angle with deionized water. Afterward, further qualitative (scratch test) and a quantitative adhesion assessment (peel test) were performed. Both scratch test and peel strength indicated that oxygen plasma treatment using reactive ion etching equipment is the most promising approach for promoting the adhesion between polyimide films.

## 1. Introduction

In the last decades, research in the field of flexible electronics has focused on aromatic polyimides (PIs), which present excellent mechanical and electrical properties. In addition, PIs exhibit good thermal and chemical stability, thus PIs are very attractive for the fabrication of flexible sensors [1,2,3,4,5,6] and medical devices [7,8]. For such applications, PI films are used as a substrate, electrical insulator, or matrix. However, this material is chemically inert and presents a smooth surface. Therefore, their proper adhesion with other materials requires specific surface treatments [9,10,11].

Much research has focused on improving the adhesion of metal layers on PI films. Plasma treatment is one of the most used treatments to increase the wettability of PI films. These treatments graft functional groups on the polymer surface, increasing its surface energy [9,12]. Plasma treatments can be used alone [13,14,15,16,17], combined with coupling agents [18], or with polymerization grating [19,20]. Another method for surface activation of PI films is based on alkaline solutions. Here, the reaction between the polymer and the solution yields to the opening of the imide ring. For metal deposition, this procedure is combined with proper ion exchange and reduction reactions [21,22,23]. In a similar fashion, amine solutions can also promote metal adhesion on PI films [11].

Interestingly, the adhesion of PI on PI layers has not been addressed as much as the adhesion of metal layers on PI. Still, this is an important issue in applications where PI films are the insulator material. For example, in neural probes [7,8] two PI layers encapsulate the device. Here, good adhesion between PI films is necessary for long-term stability. One approach for adhesion between PI layers is the partial curing of the polymer. This method is commonly used to produce thick PI layers [6,24]. Yet, this is not suitable if the device fabrication requires multiples metal/PI layers. Fur such structuring, the PI layers are fully cured before the deposition of the next layer. Thus, some sort of surface modification is required to promote the adhesion of PI to PI after curing.

The reported surface treatments are aimed to promote the adhesion of metal layers on PI, although they have potential use for improving the adhesion between PI layers. For example, plasma treatments can improve the adhesion of PI films [12] or fibers on epoxy resin [25,26] or PI precursor [9]. Treatments based on alkaline solutions, such as KOH or NaOH can be used to join two layers of polyimide [27] or PI fibers with epoxy [28]. Finally, amine-based treatments can also improve the bonding of PI films or fibers with other polymers [29,30].

The current literature has not addressed the comparison of different surface treatments for adhesion enhancement between PI films. However, scarcely related works about the adhesion of other materials to PI films can be found. For example in [31], microwave and DC-glow oxygen plasma were compared for the adhesion of chromium-copper on PI. The work of Bouhamed et al. [12] compared oxygen plasma and surface cleaning by isopropanol for adhesion between PI and epoxy composites.

The contribution of this work is the comparison of different methods for surface modification of polyimide films based on oxygen plasma treatments, alkaline, and amine solutions. The work is focused on their practical application, evaluating the adhesion force between two PI films separated by an intermediate gold layer. The first PI layer is cured before application of further material layers, as this condition represent the actual scenario for the fabrication of electronic devices. The first treatment is based on microwave oxygen plasma and the second on oxygen reactive ion etching plasma. The study comprises two oxygen plasma treatments as the adhesion may depend on the type of plasma [31]. The third treatment is based on aqueous solutions of KOH and HCl. A fourth treatment used a polyfunctional amine solution in a water/isopropanol mixture. Initially, several conditions were proposed for each method. After water contact angle measurements, one condition per method was selected. Further evaluation was performed with a qualitative scratch test, comparing the adhesion achieved. For a quantitative assessment, a 180° peel test was performed using specific samples. The results provide an interesting guide for further works related to PI film-based devices.

## 2. Materials and Methods

### 2.1. Materials

The PI prepolymer used in this work was the U-Varnish S (UBE Europe GmbH, Düsseldorf, Germany) with 20 wt.% polyamic acid content. As substrate for the PI films, a 100 mm diameter, 525 μm thick silicon wafer was used. The adhesion promoter 3-Aminopropyltriethoxysilane (APTES) from Sigma-Aldrich Chemie GmbH (Munich, Germany) guaranteed the adhesion of the PI films to the silicon wafer. For alkaline treatments, KOH 50% (MicroChemicals GmbH, Ulm, Germany) and HCl 37% (MicroChemicals GmbH) were used. For the polyfunctional amine treatment, branched Polyethylenimine (PEI) with an average molecular weight of ~800 g/mol was obtained from Sigma-Aldrich Chemie GmbH.

### 2.2. Fabrication of Polyimide Films for Surface Modification

The first step for the fabrication of the PI films required an aqueous solution (0.1 vol.%) of APTES. The adhesion promoter was spin-coated at 4000 RPM on the silicon wafer and dried for two min at 120 °C on a hotplate. Immediately after, the PI precursor was spin-coated at 3000 RPM for 60 s. This material layer was cured in a vacuum hotplate model RSS-HC (UniTemp GmbH, Pfaffenhofen an der Ilm, Germany) following the step temperature program indicated by the manufacturer, with a peak temperature of 450 °C. The resulting material was a polymer film with ~5 μm thickness.

### 2.3. Surface Treatments of Polyimide Films

Oxygen plasma was selected as a treatment for PI films since it is expected to graft functional groups on the PI surface. Two different devices were used for plasma treatments. The first equipment is mainly used for cleaning surfaces and the second is dedicated to dry etching. For each tool, a total of nine treatment conditions were proposed (see Table 1). A Tepla 400 Microwave Plasma System machine (Technics Plasma GmbH, Kirchheim bei München, Germany) was used for the first batch of PI films. The variables for this batch were the power (P) level and the treatment time (t), the gas flow was fixed to 500 mL/min. The second batch was treated with the STS Multiplex ICP Reactive Ion Etcher (RIE, Surface Technology Systems Ltd., Newport, UK), with a combination of three levels of bias power (BP) and three treatment times. The coil power (800 W), oxygen flow (40 sccm), and pressure (5 mTorr) remained constant.

The third batch used a combination of KOH and HCl solutions to help the adhesion of a second PI layer [27]. The PI films were immersed in an aqueous solution of 1 M KOH for 2, 5, and 10 min, at a fixed temperature (T) of 50 °C. The KOH solution was mechanically stirred during the procedure. After the immersion in the KOH solution, the samples were thoroughly washed with deionized water. Subsequently, the PI films were immersed in an aqueous solution of HCl (0.2 M) for 5 min at room temperature. Finally, the samples were again washed with deionized water and dried at room temperature. The last batch of coated wafers was immersed in a water/isopropanol PEI solution [32]. An amount of 2 wt.% PEI was dissolved in a combination of isopropanol and deionized water (1:1 ratio). The samples were immersed in this solution for 2, 5, and 10 min, at a constant temperature at 70 °C. Afterward, samples were washed in deionized water and dried at room temperature. Table 2 shows the treatment conditions for alkaline and polyamine treatments. Measurement of PI thickness before and after all treatments was carried out using an F20-EXR interferometer (Filmetrics Europe GmbH, Unterhaching, Germany).

### 2.4. Contact Angle Measurements

Given the number of variations of each treatment, the water contact angle was used as a simple tool to assess the wettability of PI samples after treatments. Such changes can be correlated to the chemical modification of the PI films. This procedure helped in the selection of a condition for each treatment method. A total of 24 samples were characterized, nine for microwave plasma, nine for RIE plasma, six for KOH, and six for PEI. The measurements were made with the drop shape analyzer DSA II (Krüss, Hamburg, Germany), using deionized water drops of 2.5 μL and a water flow of 50 μL/min. A total of five drops were placed on top of each film in different places. The angle was obtained automatically using the fitting software of the drop analyzer. From this analysis, the treatment conditions with the lowest contact angle of each wafer batch were selected for further adhesion tests, given a total of four treatments.

### 2.5. Fabrication of Scratch Test Samples

For the qualitative evaluation of the surface treatments, a series of bilayer PI films were manufactured. The upper PI layer of the samples was structured as circular shapes. For the fabrication, a first PI coating on a silicon wafer was produced following the method described in Section 2.2 The cured PI layer was then treated using one of the four methods selected by the contact angle method. Immediately, a second PI precursor layer was spin-coated and cured using the same speed and temperature as for the first PI coating. The second PI layer was structured using a 10 μm thickness AZ9260 photoresist layer (MicroChemicals GmbH, Ulm, Germany) and etched using O_2_/CF_4_ gas with the STS Multiplex ICP Reactive Ion Etcher. The photoresist was then removed using AZ 100 Remover. The structuring produced circles of 750 μm diameter on the top PI layer. The circle structures were afterward scratched by hand using a needle. This process was performed under an optical microscope as a simple method to evaluate the adhesion between the polymer films. A total of 10 wafers were fabricated for this test, two for each treatment and two with non-treated PI layers as a reference.

### 2.6. Fabrication of Peel Test Samples and Characterization

**Figure 1:**
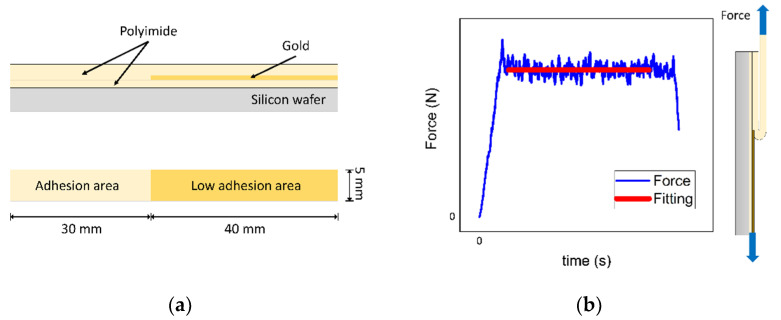
Peel test for adhesion between two PI films using Gold as a peel start: (**a**) Schematic view of the fabricated sample; (**b**) Method for computing the peel force, the lower PI film is fixed and the upper one is pulled up.

The quantitative assessment of the adhesion between PI films was performed with samples designed for a 180° peel test (see Figure 1a). The samples were fabricated by producing a first PI film (~5 μm thick) on a silicon wafer using the method described in Section 2.2 This PI film was then coated with a 100 nm thick gold layer, deposited by sputtering. The gold coating is aimed to help initiate the peeling process, due to the poor adhesion of the PI film with the gold film. The gold layer was structured using AZ1518 photoresist (1.8 μm thickness) and etched with iodine solution. As a result, six gold rectangle structures of 40 mm length and 5 mm wide were obtained. After the gold structuring, the exposed PI layer was treated using one of the four methods selected by the contact angle method. The second PI precursor layer was immediately spin-coated at 1000 RPM for 60 s and thermally cured, producing a ~10 μm thick PI film. The upper PI coating was structured with a 20 μm photoresist layer (AZ9260) and etched using O_2_/CF_4_ gas with reactive ion etching. The photoresist was removed using AZ 100 Remover. The resulting structures were six PI rectangles of 70 mm length and 5 mm width, as represented in Figure 1a. Finally, the wafer was diced to fit in a specific holder designed in CAD and fabricated using a 3D printer. A total of two wafers for each treatment were fabricated. For reference, two wafers without treatment were also fabricated.

Peel samples were placed in a Condor 100 bond tester (XYZTEC bv, Panningen, Netherlands) with a pull cell of 20 N. The parameters for the peel test were a crosshead speed of 500 μm/s, displacement of 50 mm, and a returning time of 2 s. The time of the peel experiment was 102 s. The force was obtained by fitting a constant value to the stable force region of each plot as represented in Figure 1b. This analysis was done in MATLAB (The MathWorks, Inc., Natick, MA, USA), using the least-squares method and omitting the first and last 15 s of each run.

## 3. Results

### 3.1. Wettability and Surface Energy of PI Films

The average contact angle of the pristine PI films was ~68°, indicating some degree of hydrophilicity. All treatments produced a reduction of contact angle, suggesting a modification of the PI surface. Measurements of thickness before and after the treatments indicated that the RIE plasma produced significant etching. The conditions of BP = 50 W and t = 1 min produced a thickness reduction of ~0.9 μm. The method with KOH solution also produced a noticeable etching (~0.4 μm) after 10 min treatment.

**Figure 2:**
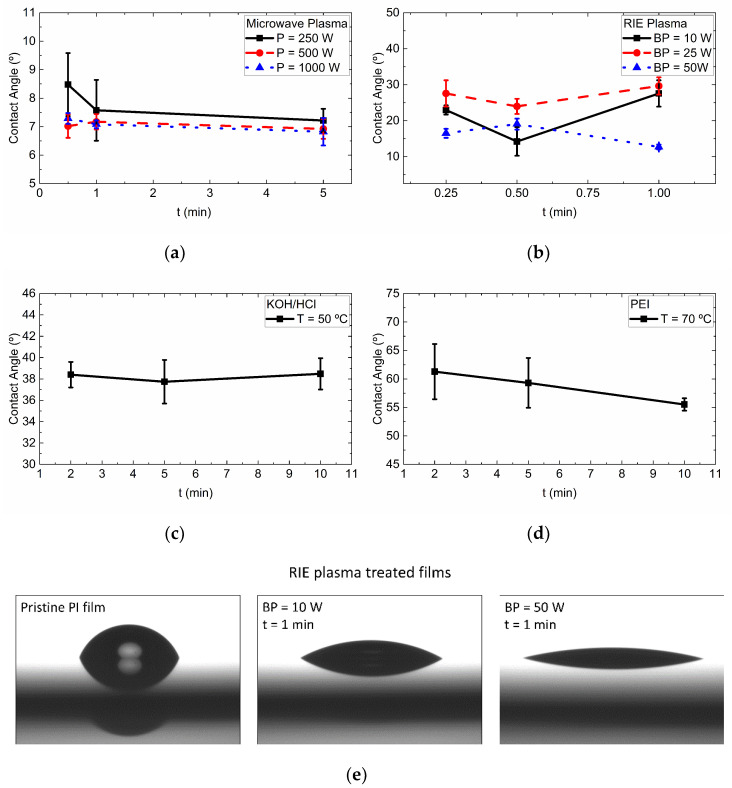
Water contact angle of treated PI films: (**a**) Microwave Plasma; (**b**) RIE Plasma; (**c**) KOH/HCl; (**d**) PEI solution; (**e**) Examples of water drops on RIE-plasma treated films.

The first batch of PI films was treated with microwave plasma (Figure 2a) and presented the highest increment in hydrophilicity. The samples exhibited a monotonic reduction of the contact angle as a function of time. For the condition of 250 W, the contact angles show a difference of ~15% between samples treated for 0.25 min and 5 min. The contact angle of films treated with 500 W and 1000 W, exhibited less than 6% difference between 0.25 min and 5 min treatments. The lowest contact angle for this treatment (6.8°) was achieved with the conditions of P = 1000 W and t = 5 min. The samples treated with the RIE plasma (second batch, Figure 2b) exhibited a non-monotonic behavior as a function of the time. For samples with a BP of 10 W or 25 W, a time of 0.5 min produced their lowest contact angle. On the other hand, the treatment with 50 W showed the opposite behavior. Here, the highest value is obtained at 0.5 min and the lowest (12.7°) is achieved with 1 min treatment.

Regarding the wet chemistry methods, the PI films immersed in the KOH solution (Figure 2c) produced similar contact angles (~38°) for all treatment times. The largest difference between them is ~2%. Although the contact angle is similar, the 10 min treatment was selected, since it is expected that longer time treatments produce higher adhesion, due to a more deeply modified layer [27] and higher surface roughness [28]. The last batch of PI films was treated with the PEI solution. The PI films exhibited the lowest modification of the contact angle (Figure 2d) of all treatments. The maximum reduction of contact angle is 20% lower than pristine PI. For this treatment, the contact angle showed a monotonic reduction as the treatment time was increased. The lowest value (55.5°) was obtained after 10 min immersion.

After comparing the contact angle for all treatment conditions, the next investigation was the calculation of the surface free energy of the PI films treated by the conditions proposed in Table 3.

**Figure 3:**
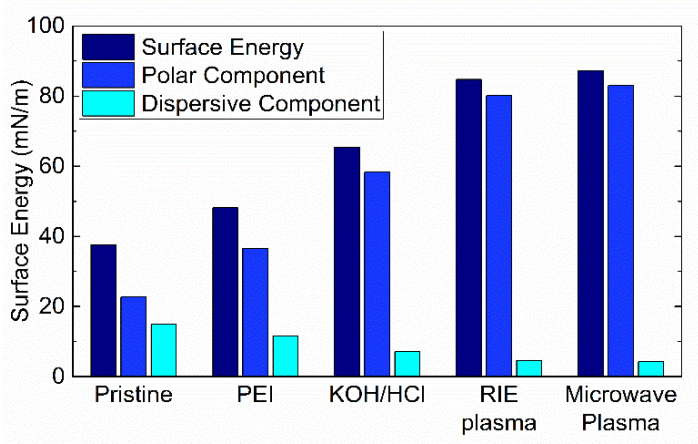
Surface free energies of pristine and treated PI films.

Since an increase in the surface energy of treated samples is expected, further calculation of the surface energy (σ_S_) was performed measuring the contact angle with DMSO and isopropanol (see details in Appendix A). The extracted values for surface energy and their polar (σ_S_^P^) and dispersive (σ_S_^D^) components are shown in Figure 3. For pristine PI, the surface energy is σ_S_ = 37.6 mN/m. This surface energy agrees with previously reported data [12,16]. The polar component and dispersive component of pristine PI films are σ_S_^P^ = 22.7 mN/m and σ_S_^D^ = 14.8 mN/m, respectively. The treated samples exhibited a reduction of the dispersive component of surface energy. In contrast, the polar portion of the surface energy was significantly increased. In the case of treatments with PEI (σ_S_ = 48.1 mN/m) and KOH (σ_S_ = 65.4 mN/m), the increment of surface energy can be attributed to the formation of polyamic amide [30] and polyamic acid [33] on the PI surface, respectively. The treatments based on plasma are expected to generate polar functional groups on the PI, increasing the surface polarity and improving the total surface energy [12,16,25]. The highest surface energy was achieved with the microwave plasma treatment (σ_S_ = 87.3 mN/m), the RIE plasma produced a 3% lower value (σ_S_ = 84.7 mN/m). This increment is ~2.3 times the surface energy of non-treated PI films.

### 3.2. Scratch Test of Polyimide Films

The scratch test was used as a quick evaluation of the effectiveness of selected treatment conditions (Table 3) to promote the adhesion between two PI layers. The samples were fabricated from two PI layers spin-coated silicon wafers. The first PI layer was treated using the proposed conditions before applying the second layer. The upper layer was then structured as 750 μm diameter circles.

**Figure 4:**
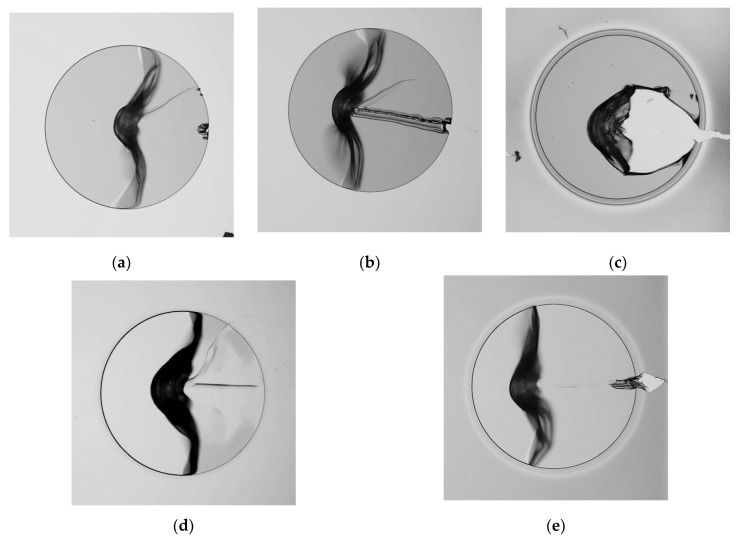
Scratch test of PI films: (**a**) Pristine PI; (**b**) Microwave Plasma; (**c**) RIE Plasma: (**d**) KOH/HCl: (**e**) PEI solution.

The qualitative test of the PI bilayer samples was performed by removing the upper PI layer with a needle. Light microscope pictures of representative scratch samples are presented in Figure 4. The samples corresponding to the pristine PI (Figure 4a) were easy to remove, even allowing the complete removal of the top layer. The exposed bottom layer of these samples showed a smooth surface. The samples treated with microwave oxygen plasma are shown in Figure 4b. The PI coating was also easy to remove and the exposed surface was similar to the samples without treatment. This indicates that a low contact angle alone is not enough to ensure the adhesion between the PI layers. This is similar to what was observed in [31], where samples treated by microwave plasma did not show adhesion enhancement with sputtered chromium-copper. The plasma treatment performed by RIE (Figure 4c) produced films that could not be removed. Both the lower and upper PI layers were torn. In the case of treatment with KOH and HCl solutions (Figure 4d), the films could be peeled, although more difficult than the microwave plasma samples. Some shadows on the surface of the first layer suggested a degree of adhesion and surface modification. The last treatment consisting of immersion in PEI solutions is presented in Figure 4e. Here, the upper layer could be removed using similar force to that applied to pristine films or the samples treated with microwave plasma. No noticeable surface changes were observed in PEI-treated layers.Although the scratch test provides a good idea of the adhesion between PI layers, it does not provide an objective comparison of the force required to separate the layers. For this reason, a peel test using specific samples was performed to avoid bias or errors from the side of the evaluator.

### 3.3. Peel Strength between PI Films

**Figure 5:**
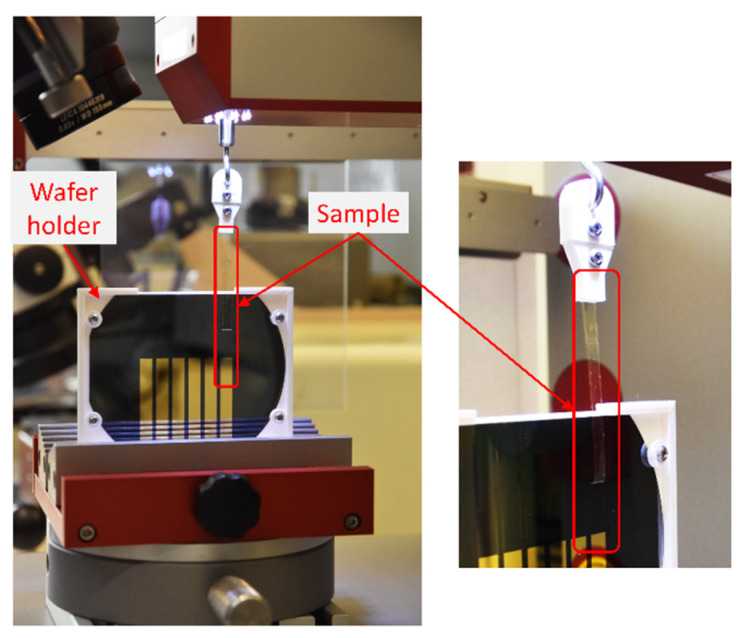
Example of a peel test specimen and setup.

The samples for the 180° peel test of PI films consisted of polymer strips of 70 mm long and 5 mm width. The specimens have a treated area for the adhesion (30 mm long × 5 mm width) and an intermediate gold layer as the non-bonded section (40 mm long × 5 mm width) due to the low adhesion of the PI with the gold layer. The samples were fabricated on a silicon wafer, obtaining six peel samples (strips) per wafer. An example of the experimental setup is shown in Figure 5.

**Figure 6:**
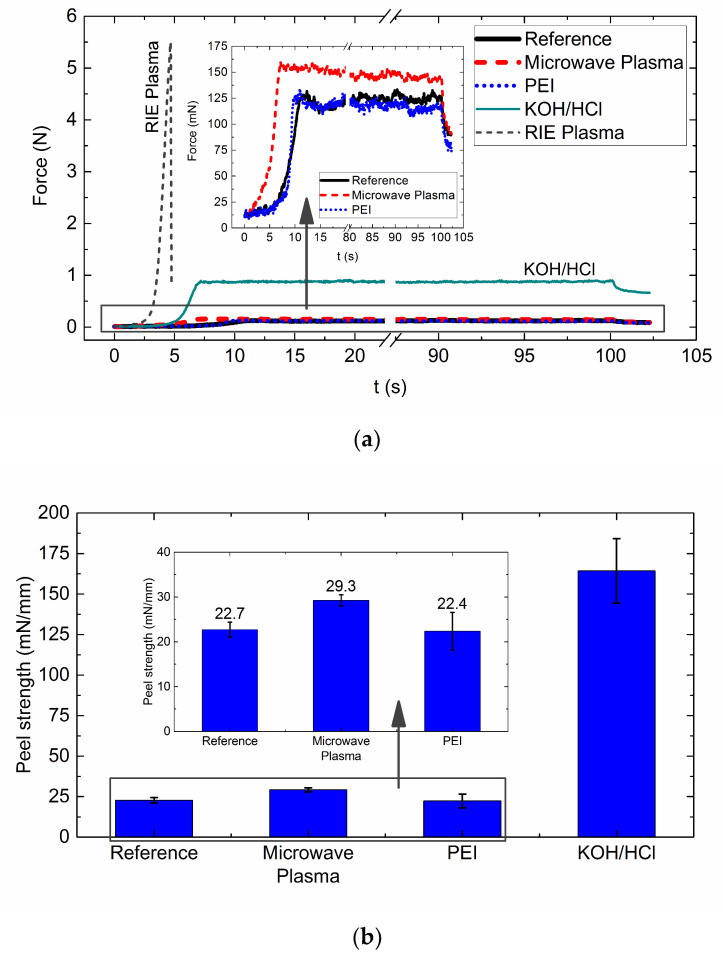
Peel test of results of PI films: (**a**) Representative force plot of samples; (**b**) Average peel strength.

Two wafers per treatment and two wafers without treatment were evaluated with the 180° peel method. A total of 12 measurements was done for each treatment and for the reference material. The peel strength of each strip specimen was obtained by fitting a constant value to the stable force region of each data plot. This value was then divided by the sample width (5 mm) to compute the peel strength. Representative force curves of the samples are presented in Figure 6a, an inset is included for the smaller force curves.

The average peel strength of non-treated PI films was 22.7 mN/mm and serves as a reference for all treatments. This result agrees with the peel strength of 23 mN/m and 25 mN/m, previously reported in [27] and in [24]. This value, however, is very low compared to the peel strength (1.06 N/mm) reported by Ree et al. [34], which can be attributed to the use of different polyimide or fabrication conditions. The same can be said for adhesion of metal layers by sputtering [11,14,31] on PIs, where no PI treatment is required to achieve higher peel strength values (250–400 mN/mm).

The treatment with microwave plasma showed a peel strength of 29.3 mN/mm. This value is 29% higher than pristine PI film, indicating some degree of adhesion. Such level of improvement was not possible to determine with scratch test only. Although no PI on PI adhesion by microwave oxygen plasma is available in the literature, this method has been used for adhesion improvement of metal layers on PI. For example, atmospheric plasma used in [16], indicated that peel strength of electroless deposited Cu can be enhanced from 0.2 N/mm to 0.35 N/m. Moreover, the use of coupling agents [18] can even increase the peel strength up to 0.785 N/mm.

The average value for PI films treated with PEI did not show improvement of peel strength compared to the pristine polymer. The average peel strength is 22.4 mN/mm, which agrees with the noticed effort in the qualitative scratch test. This result is contrary to the observed by Park et al. [11], where amine solutions yielded adhesion increment from 245 mN/mm to 638 mN/mm for sputtered Cu. This may be attributed to the effect of treatment conditions, as well as the use of different modifiers [30]. The use of KOH solution followed by HCl produced a significant adhesion improvement compared to previous treatments. The samples required ~7 times the force of pristine films to remove the upper PI layer (peel strength = 164 mN/mm). This increment of adhesion between PI layers may explain the surface changes observed on the surface of the bottom PI layer during the scratch experiment. By comparison, the reported peel strength of PI film on PI modified using KOH by Lee et al. [27] is 0.95 N/mm. In the case of electroless deposited Cu, the peel strength can be increased from 0.29 N/mm up to 1.66 N/mm by including an alkaline permanganate treatment after KOH, which increases the surface roughness of PI [22].

The samples treated with RIE oxygen plasma could not be peeled. The force required is above the mechanical resistance of PI films. This result agrees with the previous qualitative test, showing that the highest adhesion is achieved with the RIE oxygen plasma. This adhesion level has been reported by Ree et al. [34] in a study of adhesion between PI films, and PI films with others substrates. They reported that non-peelable PI on PI films is achieved when the first PI layer is treated by plasma ashing. The results indicate that RIE plasma is the best procedure to promote the adhesion between PI layers. In addition, the method is easy to include in the manufacturing steps of flexible electronic devices. The comparison of average peel strength of all treatments (for peelable samples) is shown in Figure 6b).

## 4. Discussion of the Adhesion Mechanisms between PI Films

The best adhesion between the PI layers was achieved using RIE oxygen plasma treatment, using the conditions of BP = 50 W and 1 min long. This treatment also produced a reduction in the contact angle, from 67.6° of pristine samples to 12.7°. RIE plasma also produced the highest amount of etching, yielding a reduction of 0.92 μm thickness in PI films. For easy comparison with other treatments, Table 4 summarizes the parameters used for treatment and the average peel strength. For completeness, the average values of contact angle, surface energy and etching are also included. The treatments are sorted from lowest to highest achieved adhesion between the PI layers.

From the data in Table 4, it can be noticed that the two treatments that produced a significant improvement of the adhesion are also the treatments that produced etching of PI films. These two treatments also promoted a reduction in the contact angle and an increment of the surface energy, only surpassed by the microwave plasma method.

The improvement in adhesion of the PI films produced by RIE oxygen plasma can be attributed to changes in the chemical composition and surface roughness of the films. A more in-depth study of polymer surfaces is beyond the scope of this work. This section provides a brief description of the adhesion mechanisms previously discussed by other authors. For example, PI fibers [9,25,26] and PI films [12,16,35] treated by oxygen plasma present an increase in the O/C ratio, observed by XPS. This change is correlated to a higher concentration of C–O and C=O bonds produced by oxygen plasma. A possible modification of the PI structure is the opening of the imide ring. This yields the formation of polyamic acid or PI precursor [25,26]. The formation of secondary functional is also one possibility [9], as radicals formed during the treatment can produce secondary groups such as –OH or –COOH. The change in the surface chemistry can result in an increment in the wettability of the PI films or fibers [25,26,35]. This modification can be useful to promote the chemical interaction with some resins or a PI precursor. However, treatments applied beyond a certain time or power range can produce an over-etching. This condition leads to the removal of the outer chemically active layer of the PI. This over-etching causes a reduction of its wettability [9,35]. This phenomenon may explain the non-monotonic behavior of the samples treated with RIE oxygen plasma.

The surface roughness of PI films and fibers is also changed by plasma treatments due to the etching [9,35], crosslinking and polymer chain scission [15,16]. Such modification has an important contribution to the adhesion strength of the material. The increment of the surface area leads to higher infiltration of molecules. For example in [26], PI fibers with higher surface roughness (obtained by AFM) presented improved interfacial shear strength with epoxy resin. Nevertheless, very aggressive treatments can decrease the surface roughness due to excessive etching. Since the samples treated by RIE plasma presented noticeable etching and an increment of wettability, the changes in the chemical structure of PI films and the increment in the surface roughness are probably the main driving mechanisms of the adhesion.

## 5. Conclusions

Four methods for surface modification were used to evaluate their effectiveness to improve the adhesion between two polyimide layers. The first method is based on microwave oxygen plasma and the second method used reactive ion etching oxygen plasma. The third method consisted of a combination of aqueous solutions of KOH and HCl, and the last method used polyethylenimine in a mixture of water and isopropanol.

From contact angle measurements, one condition variant for each method was selected based on its highest wettability. The microwave plasma treatment produced the highest wettability on polyimide surfaces. Both microwave and RIE plasmas enhanced ~2.3 times the surface energy of polyimide films. The use of polyethylenimine solution produced the lowest wettability and lowest enhancement of surface energy.

A series of 5 μm thick bilayer polyimide films were used for qualitative assessment of the adhesion by scratch test. The first layer of these samples was modified using one of four selected treatments and the second polyimide layer was removed by hand with a needle. This experiment showed that it was not possible to remove the PI layers treated with RIE plasma. All other treatments produced PI films that could be removed.

For a quantitative comparison between treatments, samples of 10 μm thick polyimide for a 180° peel test were fabricated. The treatments with microwave plasma showed a 29% increment of peel strength compared to untreated polymer, whereas the polyethylenimine treatment did no show improvement. The treatment based on KOH/HCl produced an increment of 7 times peel strength compared to pristine polyimide. The samples treated with RIE plasma were not able to peel, indicating that it is the best approach for interlayer adhesion of polyimide films.

## Figures and Tables

**Table 1:** Parameter combinations for the oxygen plasma treatments.

Device	Time (Min)	Power (Watts)
Microwave Plasma	0.5, 1.0, 5.0	250, 500, 1000
ICP RIE	0.25, 0.5, 1.0	10, 25, 50

**Table 2:** Time variations for the wet chemistry treatments.

Solution	Time (Min)	Temperature (°C)
KOH	2, 5, 10	50
PEI	2, 5, 10	70

**Table 3:** Selected treatment conditions for scratch and peel test of PI films.

Treatment	Conditions
Microwave Oxygen Plasma	P = 1000 W, t = 5 min
RIE Oxygen Plasma	BP = 50 W, t = 1 min
KOH/HCl	KOH: T = 50 °C, t = 10 min
PEI	T = 70 °C, t = 10 min

**Table 4:** Summarizing of characterization results of PI films.

Treatment	Conditions	Peel Strength (mN/mm)	Contact Angle (°)	Surface Energy (mN/m)	Etching (μm)
Pristine	-	22.7 ± 1.65	67.6 ± 2.78	37.6	-
PEI	T = 70 °C t = 10 min	22.4 ± 4.19	55.5 ± 1.09	48.1	Neglectable
Microwave plasma	P = 500 W t = 5 min	29.3 ± 1.24	6.82 ± 0.48	87.3	Neglectable
KOH/HCl	T = 50 °C t = 10 min	164.3 ± 19.9	38.4 ± 2.04	65.4	0.40 ± 0.024
RIE Plasma	BP = 50 W t = 1 min	Non-peelable	12.7 ± 0.69	84.7	0.92 ± 0.04

**Figure A1:**
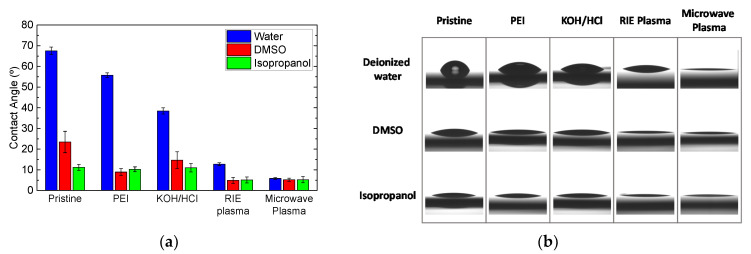
Contact angle of PI films: (**a**) Average values for three solvents on pristine and treated films; (**b**) Example of solvent drops on PI films.

## Appendix A. Calculation of Surface Free Energy of PI Films

The surface free energy of pristine and treated PI films with the conditions of Table 3 was determined using the sessile drop method. The surface energy (σ) is related to the contact angle and is expressed as the sum of polar (σ^P^) and dispersion (σ^D^) components. Three liquids were used for wetting the polymer surface, deionized water, DMSO, and isopropanol. The surface tension of each liquid (σ_L_) and their polar (σ_L_^P^) and dispersive (σ_L_^D^) components are listed in Table A1. The contact angle was measured with the drop analyzer DSA II by placing five drops on the polymer surface. One sample without treatment and one wafer per each selected treatment was used in this evaluation.

**Table A1:** Surface tension and components of solvents [16,36,37].

Liquid	σ_L_ (mN/m)	σ_L_^P^ (mN/m)	σ_L_^D^ (mN/m)
Water	72.8	51.0	21.8
DMSO	44.0	8.0	36.0
Isopropanol	23.0	3.5	19.5

The Owens-Wendt expression [38] can be used to calculate the dispersive (σ_S_^D^) and polar components (σ_S_^P^) of polymer films. In its linear form, this model is defined as,

0.5σ_L_ (1 + cosθ)/(σ_L_^P^)^1/2^ = (σ_S_^D^)^1/2^ (σ_L_^D^/σ_L_^P^)^1/2^ + (σ_S_^P^)^1/2^, (A1)

where θ is the contact angle of the liquid drop on the solid surface and the total surface tension of the liquid is represented by σ_L_. The dispersive and polar components of the surface energy are σ_L_^D^ and σ_L_^P^, respectively. Since the known values are σ_L_, σ_L_^D^ and σ_L_^P^, the contact angle of at least two liquids is required to determine the dispersive (σ_S_^D^) and polar (σ_S_^P^) components for the PI films (σ_S_ = σ_S_^D^ + σ_S_^P^). For the extraction of the surface energies values, the Equation A1 was plotted as the lineal function y = mx + b, where y = 0.5σ_L_ (1 + cosθ)/(σ_L_^P^)^1/2^ and x = (σ_L_^D^/σ_L_^P^)^1/2^. The linear fit to the data points allowed calculating the slope m = (σ_S_^D^)^1/2^ and the intercept b = (σ_S_^P^)^1/2^.

The average contact angles for the three liquids are presented in Figure A1a. The treated PI films exhibited lower contact angles with all solvents compared to the pristine material. The most noticeable change is the angle reduction with deionized water with the microwave plasma treatment. The samples treated using the PEI solution showed the highest contact angle with water. Regarding DMOS and isopropanol, the samples treated by RIE and microwave plasma showed the lowest contact values. The pictures in Figure A1b show the variation of the contact angle for some drop samples.

## References

[1] Boll D., Schubert K., Brauner C., Lang W. (2014). Miniaturized Flexible Interdigital Sensor for In Situ Dielectric Cure Monitoring of Composite Materials. IEEE Sens. J..

[2] Gosh M.K., Mittal K.L. (1996). Polyimides: Fundamentals and Applications.

[3] Chou C.-Y., Liu H.-S., Liou G.-S. (2016). Highly transparent silver nanowire–polyimide electrode as a snow-cleaning device. RSC Adv..

[4] Yang T., Yu Y.Z., Zhu L.S., Wu X., Wang X.H., Zhang J. (2015). Fabrication of silver interdigitated electrodes on polyimide films via surface modification and ion-exchange technique and its flexible humidity sensor application. Sens. Actuators B Chem..

[5] Hubner M., Lepke D., Hardi E., Koerdt M., Herrmann A.S., Lang W. (2019). Online Monitoring of Moisture Diffusion in Carbon Fiber Composites Using Miniaturized Flexible Material Integrated Sensors. Sensors.

[6] Engel J., Chen J., Liu C. (2003). Development of polyimide flexible tactile sensor skin. J. Micromech. Microeng..

[7] Schander A., Stemmann H., Kreiter A.K., Lang W. (2018). Silicon-Based Microfabrication of Free-Floating Neural Probes and Insertion Tool for Chronic Applications. Micromachines.

[8] Schander A., Strokov S., Stemmann H., Tebmann T., Kreiter A.K., Lang W. (2019). A Flexible 202-Channel Epidural ECoG Array With PEDOT: PSS Coated Electrodes for Chronic Recording of the Visual Cortex. IEEE Sens. J..

[9] Lin F., Li W., Tang Y., Shao H., Su C., Jiang J., Chen N. (2018). High-Performance Polyimide Filaments and Composites Improved by O_2_ Plasma Treatment. Polymers.

[10] Qu H., Wang Z., Cang D. (2019). Flexible Bandpass Filter Fabricated on Polyimide Substrate by Surface Modification and In Situ Self-Metallization Technique. Polymers.

[11] Park Y.J., Yu D.M., Ahn J.H., Choi J.-H., Hong Y.T. (2012). Surface modification of polyimide films by an ethylenediamine treatment for a flexible copper clad laminate. Macromol. Res..

[12] Bouhamed A., Kia A.M., Naifar S., Dzhagan V., Müller C., Zahn D.R.T., Choura S., Kanoun O. (2017). Tuning the adhesion between polyimide substrate and MWCNTs/epoxy nanocomposite by surface treatment. Appl. Surf. Sci..

[13] Miyauchi K., Yuasa M. (2013). A study of adhesive improvement of a Cr–Ni alloy layer on a polyimide surface by low pressure gas plasma modification. Prog. Org. Coat..

[14] Usami K., Ishijima T., Toyoda H. (2012). Rapid plasma treatment of polyimide for improved adhesive and durable copper film deposition. Thin Solid Films.

[15] Lin Y.S., Liu H.M. (2008). Enhanced adhesion of plasma-sputtered copper films on polyimide substrates by oxygen glow discharge for microelectronics. Thin Solid Films.

[16] Park S.J., Lee H.Y. (2005). Effect of atmospheric-pressure plasma on adhesion characteristics of polyimide film. J. Colloid Interface Sci..

[17] Inagaki N., Tasaka S., Hibi K. (1994). Improved adhesion between plasma-treated polyimide film and evaporated copper. J. Adhes. Sci. Technol..

[18] Lai Y.-H., Chandra Sil M., Chen C.-M. (2021). Surface composite engineering of polyimide to create amine functionalities for autocatalytic metallization. Appl. Surf. Sci..

[19] Yu Z.J., Kang E.T., Neoh K.G. (2002). Electroless plating of copper on polyimide films modified by surface grafting of tertiary and quaternary amines polymers. Polymer.

[20] Inagaki N., Tasaka S., Masumoto M. (1996). Improved Adhesion between Kapton Film and Copper Metal by Plasma Graft Polymerization of Vinylimidazole. Macromolecules.

[21] Yu W.X., Hong L., Chen B.H., Ko T.M. (2003). A study on the interfacial composition of the electroless-copper-plated BPDA-PDA polyimide sheet. J. Mater. Chem..

[22] Wang Z., Furuya A., Yasuda K., Ikeda H., Baba T., Hagiwara M., Toki S., Shingubara S., Kubota H., Ohmi T. (2002). Adhesion improvement of electroless copper to a polyimide film substrate by combining surface microroughening and imide ring cleavage. J. Adhes. Sci. Technol..

[23] Wu P.-Y., Lin C.-H., Chen C.-M. (2017). Study of Surface Metallization of Polyimide Film and Interfacial Characterization. Metals.

[24] Brown H., Yang A., Russell T., Volksen W., Kramer E. (1988). Diffusion and self-adhesion of the polyimide PMDA-ODA. Polymer.

[25] Sun X., Bu J., Liu W., Niu H., Qi S., Tian G., Wu D. (2017). Surface modification of polyimide fibers by oxygen plasma treatment and interfacial adhesion behavior of a polyimide fiber/epoxy composite. Sci. Eng. Compos. Mater..

[26] Wen Y., Meng X., Liu J., Yan J., Wang Z. (2016). Surface modification of high-performance polyimide fibers by oxygen plasma treatment. High Perform. Polym..

[27] Lee K.W., Kowalczyk S.P., Shaw J.M. (1991). Surface modification of BPDA-PDA polyimide. Langmuir.

[28] Tian G., Chen B., Qi S., Niu H., Han E., Wu D. (2015). Enhanced surface free energy of polyimide fibers by alkali treatment and its interfacial adhesion behavior to epoxy resins. Compos. Interfaces.

[29] Xie F., Zhang N., Lu Z., Zhuo L., Yang B., Song S., Qin P., Wei N. (2018). Highly improved mechanical and dielectric properties of paper-based composites with polyimide chopped fiber functionalized by ethylenediamine. High Perform. Polym..

[30] Yun H.K., Cho K., Kim J.K., Park C.E., Sim S.M., Oh S.Y., Park J.M. (1997). Adhesion improvement of epoxy resin/polyimide joints by amine treatment of polyimide surface. Polymer.

[31] Egitto F.D., Matienzo L.J., Blackwell K.J., Knoll A.R. (1994). Oxygen plasma modification of polyimide webs: Effect of ion bombardment on metal adhesion. J. Adhes. Sci. Technol..

[32] Albrecht W., Seifert B., Weigel T., Schossig M., Holländer A., Groth T., Hilke R. (2003). Amination of Poly(ether imide) Membranes Using Di- and Multivalent Amines. Macromol. Chem. Phys..

[33] Kim H.J., Park Y.J., Choi J.-H., Han H.S., Hong Y.T. (2009). Surface modification of polyimide film by coupling reaction for copper metallization. J. Ind. Eng. Chem..

[34] Ree M., Park Y.H., Shin T.J., Nunes T.L., Volksen W. (2000). Self-adhesion of poly(4,4′-oxydiphenylene biphenyltetracarboximide) and its adhesion to substrates. Polymer.

[35] Kim S.H., Na S.W., Lee N.E., Nam Y.W., Kim Y.-H. (2005). Effect of surface roughness on the adhesion properties of Cu/Cr films on polyimide substrate treated by inductively coupled oxygen plasma. Surf. Coat. Technol..

[36] Zhang Z., Wang W., Korpacz A.N., Dufour C.R., Weiland Z.J., Lambert C.R., Timko M.T. (2019). Binary Liquid Mixture Contact-Angle Measurements for Precise Estimation of Surface Free Energy. Langmuir.

[37] Yarce C.J., Pineda D., Correa C.E., Salamanca C.H. (2016). Relationship between Surface Properties and In Vitro Drug Release from a Compressed Matrix Containing an Amphiphilic Polymer Material. Pharmaceuticals.

[38] Owens D.K., Wendt R.C. (1969). Estimation of the surface free energy of polymers. J. Appl. Polym. Sci..

